# Assessing aflatoxin exposure risk from imported nuts in the Jordan market

**DOI:** 10.1016/j.toxrep.2025.102190

**Published:** 2025-12-16

**Authors:** Abdalmajeed M. Alajlouni, Dima Alkadri, Mohammad S. Abu-Hardan, Amer A. Al-Sakaji

**Affiliations:** a Veterinary Pathology and Public Health Department, Jordan University of Science and Technology, Irbid 22110, Jordan; b Department of Food Science and Nutrition, Faculty of Agriculture, Jerash University, Jerash, Jordan; c Aqaba International Laboratories/BENHAYYAN, ASEZA, Aqaba 77110, Jordan

**Keywords:** Nuts, Aflatoxins, Risk Assessment, HPLC, MOE

## Abstract

This study assessed the contamination levels and health risk of aflatoxins in nuts from the markets in Jordan. A total of 180 nut samples (pistachios, almonds, walnuts, and cashews) were analyzed using high-performance liquid chromatography (HPLC) following immunoaffinity column clean-up and QuEChERS extraction. Aflatoxins were detected in 13 % of the samples, with pistachios showing the highest contamination rate. The Estimated Daily Intake (EDI) and Margin of Exposure (MOE) were calculated using deterministic risk assessment methods, based on the mean contamination levels and average nut consumption patterns in Jordan. All MOE values were below the safety threshold of 10,000, indicating a potential health risk. These findings emphasize the need for strengthened monitoring programs and regulatory actions to ensure food safety and minimize the public health risk.

## Highlights

• 25 (13 %) nut samples contain aflatoxins above the quantification limit.
• Pistachio samples showing the highest contamination rate.
• The Estimated Daily Intake (EDI) and Margin of Exposure (MOE) were calculated.
• All MOE values were below the safety threshold of 10,000.

## Graphical Abstract

**Figure:**
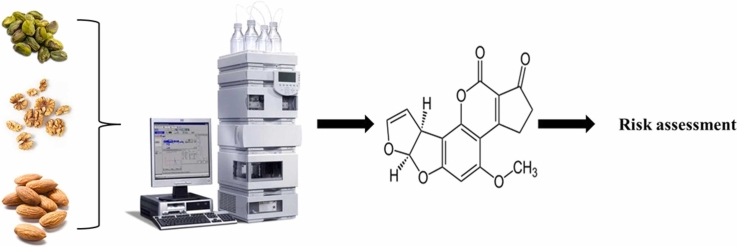

## 1 Introduction

Aflatoxins are toxic secondary metabolites naturally produced by fungi, particularly *Aspergillus flavus* and *Aspergillus parasiticus* [1]. These compounds are considered mutagenic, teratogenic, and potent carcinogens. Contamination of food and feed with aflatoxins has been a significant safety concern due to the potential health risks to humans and animals [15], [21], [22], [24]. Nuts such as peanuts, pistachios, and almonds are particularly susceptible to aflatoxin contamination, leading to serious economic losses and public health threats [16], [23]. The risk of contamination with aflatoxins can occur at any stage of nut production, including pre-harvest, harvest, post-harvest stages of production, handling, and storage [16], [2].

The EFSA [13] reported that of the major food groups contributing to aflatoxin B1 exposure, grain and grain-based products are the most frequently implicated food groups for all ages. However, it is notable that nuts belong to another major food group: legumes, nuts, and oilseeds. High aflatoxin B1 values were detected in other types of nuts, including pistachios [8], [13], [18].

In Jordan, in addition to the consumption of nuts directly, the nuts are consumed within many sweets’ recipes. The sources of nuts in Jordan are from local crops and through importation. Jordan has a hot and dry climate that represents the good conditions for the fungus to grow and produce its toxins. Besides that, the improper harvesting, transportation, and storage practices play an important role in the growth of fungus and toxin production [19]. Based on Jordanian regulations, the maximum levels of aflatoxins in nuts are differ based on the type of nut. The levels for aflatoxin B1 and total aflatoxin in pistachio are (5, 15 μg/kg), while for almond, cashew, and walnut, the limits not specified in the Jordanian standards, but they refer to the international standards, and the limits are (5, 10 μg/kg) [9], [13].

Since aflatoxins are carcinogenic compounds, the risk of consuming those toxins should be characterized. The Margin of Exposure (MOE) approach is recommended by expert groups of EFSA, the Joint FAO/WHO expert committee on Food Additives (JECFA), and the International Life Sciences Institute (ILSI) to study the genotoxicity and carcinogenicity of compounds that present in a botanical or botanical preparation [11]. MOE is a more powerful tool for studying carcinogenicity than NOAEL or LOEAL because it gives more predicted and precise information about the dose response effect to determine the point of departure and to extrapolate from this point for relevance to human exposure. The MOE is a ratio of a reference point obtained from epidemiologic or experimental data on tumor incidence, mainly from animal’s experiments, which is divided by the estimated daily intake in humans [11]. The reference point used in determining the MOE is the BenchMark Dose (BMD).

Few studies have documented the presence of aflatoxins in nuts and other food products in Jordan. Sirhan 2013 et al., analyzed 669 samples of different imported foods to Jordan. The results showed the highest concentration of aflatoxins in peanut, peanut butter, pistachio, and sesame, respectively [20]. A study conducted during 2010–2011 in Amman showed that all food samples contain aflatoxins below Jordan’s and international safety thresholds [3]. Still, the data on aflatoxin levels in food consumed by Jordanians needs more research to understand the current situation well. In addition, none of or a few of the previous studies tried to figure out the risk associated with high Aflatoxin MRL. Therefore, the objective of the current study was to quantify the aflatoxin residues in highly consumed nuts in Jordan, including pistachio, almond, walnut, and cashew and evaluate the risks associated with the consumption of contaminated nuts.

## 2 Materials and methodology

### 2.1 Sample collection

One hundred eighty samples of nuts were collected randomly from the entire retail market across Jordan throughout 2024 covering different months to account the seasonal variations. Sampling was carried out to ensure geographical representation from major cities. Shops were selected based on market size, sales volume, and consumer accessibility. The samples were (Pistachio, almond, cashew, and walnut). These products were chosen due to their high consumption and people’s habit of eating them directly or within the local sweet. Approximately 1 kg of each sample was collected from different shops. The samples were immediately transported to the laboratory in sealed bags under controlled conditions to prevent external contamination or moisture absorption. Upon arrival, samples were analyzed directly to minimize any contamination or storage related degradation.

### 2.2 Chemicals and reagents

The aflatoxin-certified reference materials used present high purity grades (>97 %) with concentrations of 20 µg/ mL and were purchased from Sigma, (France). Acetonitrile (ACN) HPLC grade, methanol HPLC grade. toluene HPLC grade, ethyl acetate, anhydrous magnesium sulfate (purity > 99.9 %), Octadecyl Endcapped Sorbent (C18 Sorbent) (UCT or equivalent), Primary Secondary Amine Sorbent (PSA bonded silica) (UCT or equivalent) were purchased from Carlo Erba (France).

### 2.3 Extraction of aflatoxins

The sample extraction and cleanup procedures were based on QuEChERS method for the sample preparation method for aflatoxins [7]. The entire sample is homogenized and ground until a fine and homogeneous powdered material is obtained. Then, 10 ± 0.1 g of each sample is transferred into a 50 mL disposable centrifuge tube and 10 mL of ultrapure water is added, followed by shaking and vortexing for 1 min. Next, 15.0 mL of ACN (acetonitrile) is added, and the mixture is shaken for 1 min and vortexed for 30 s. After that, the sample is sonicated in an ultrasonic bath for 10 min. After sonication, 1.0 ± 0.1 g of NaCl and 6.0 ± 0.1 g of anhydrous MgSO₄ are added, followed by shaking for 1 min and vortexing for 30 s. The sample is centrifuged at 3600 rpm for 2 min. After centrifugation, 8 mL of the ACN layer is transferred to a 15 mL centrifuge tube and stored in a freezer at < -20°C for 2 h ± 10 min. After freezing, the cold sample is centrifuged at 3600 rpm for 2 min.

For sample cleanup, 6 mL of the ACN layer is taken from the 15 mL graduated disposable centrifuge tube. 900 mg of anhydrous MgSO₄, 350 mg of Primary Secondary Amine sorbent (PSA), and 350 mg of Octadecyl Endcapped sorbent (C18) are added. The mixture is shaken thoroughly and then vortexed for 30 s. The sample is centrifuged at 3600 rpm for 2 min. 3 mL of the extract is transferred using a verified automatic pipette into a 50 or 100 mL round-bottom flask. The extract is evaporated to dryness using a rotary evaporator. Finally, the residue is re-dissolved in 1000 µL of HPLC-grade toluene.

### 2.4 Quantification of aflatoxins

Quantification of aflatoxins in samples was performed using an HPLC Agilent model (Waldborn, Germany) system consisting of a pump with a quaternary configuration, a vacuum degasser, a column oven, a fluorescence detector, and coupled with an auto sampler equipped with a 20 µl sample loop. The chromatographic separation was performed with a HPLC column (Agilent ZORBAX RX-SIL 4.6 ×150 mm 5-Micron, or equivalent) and was purchased from (Santa Clara, USA). The sample extracts were analyzed using 90:5:2.0: 3.0 toluene/ethylacetate/formic acid/methanol as the mobile phase. The column was kept in a column oven at 40 ºC at a flow rate of 1.5 mL/min. The injection volume was maintained at 20 µl for both the sample and standard solutions.

### 2.5 Calibration graph of aflatoxins

A standard solution of 1 µl /mL (1 ppm) was prepared by transferring 0.05 mL of mixed standard solution 20 µl / mL (ppm) to a 1 mL volumetric flask and then completing to the mark with toluene. A calibration curve of 7 concentrations (1–100 ng/mL) was used. The suitability of the calibration curve was checked each time by injection of 20 μl of one of the calibration curve standard solutions three times, and the responses should be within ± 20 % of the original response. In addition, the linearity (R^2^) was checked and it should be more than 0.98.

### 2.6 Method validation and quality control

To evaluate the reliability and quality of the results, with each batch of samples, analysis of a blank sample was performed, 10 % of the samples were analyzed in duplicate, and the Limit of Quantitation (LOQ), linearity, recovery, precision, both repeatability and within-laboratory reproducibility, were determined. For the recovery and reproducibility evaluations, standard solutions at concentrations of 5 μg/kg sample were added to untreated samples. The LOQ was established based on the recovery results of these repetitions, defined as the lowest concentration that met an acceptable recovery rate between 70 % and 120 %, along with maintaining a relative standard deviation (RSD) of 20 % or less.

### 2.7 Risk assessment of aflatoxin intake and calculation of MOE

Estimated daily intakes (EDI) were calculated takeing into account different diets, the mean concentration of aflatoxins in nut samples, a body weight of 60 kg, and mean food intakes are 7, 10, 6, 10 g/day for pistachio, cashew, almond, and walnut, respectively, [14]. The full range of BMDL10 values (i.e., lowest to highest BMDL10 value), which is 0.250 μg/kg.bw./day, was compared with the estimated daily intakes of the aflatoxins of interest resulting from the use of nuts to derive an MOE range [6]. MOE values are rounded to a single significant figure. An MOE > 10,000 is considered a low priority for risk management actions and would be of low concern from a public health point of view [11].

## 3 Results and discussion

The objective of the current paper was to assess the risk of exposure to aflatoxins as a result of consumption of nuts. Based on the chemical analysis of 180 samples of nuts, 25 samples (13 %) appeared to contain aflatoxins, and these are 11 samples of pistachio, 7 samples of cashew, 3 samples of almonds, and 2 samples of walnuts.

### 3.1 Method validation

Blank samples of the selected nuts were fortified with the aflatoxin standard solutions at 5 and 50 μg/kg levels. Table S1 exhibits the matrix-matched calibration curve equations performed for aflatoxin at levels ranging from (1−100) ng/mL and the results of recovery experiments. The recovery for the four aflatoxins (B1, B2, G1, and G2) was 70–120 %. Figure S1 shows the matrix-matched chromatogram of aflatoxins. The relative standard deviations for repeatability and intermediate precision were less than 9 % and 14 %, respectively. The LOQ of all aflatoxins was1 μg/kg (the lowest verified level), and the LOD was 0. 5 μg/kg.

### 3.2 Quantification of aflatoxins in samples

A total of 180 samples of nuts were analyzed using HPLC to detect and quantify aflatoxins. Table 1 represents the results of the aflatoxins in the samples. The selected nuts were pistachio, almond, cashew, and walnut.

**Table 1:** Mean concentration with standard deviation, EDI and MOE values of aflatoxins in samples.

Nuts type	Sample #	Aflatoxins level (µg/kg)		EDI of AF1 µg/kg.bw	MOE
		B1	Total		
Pistachio	1	1.79 ± 0.03	4.66 ± 0.02	0.000209	1197
	2	20.78 ± 0.50	22.96 ± 0.85	0.002424	103
	3	27.40 ± 1.53	29.70 ± 1.06	0.003197	78
	4	243.83 ± 10.45	257.26 ± 8.25	0.028447	9
	5	23.24 ± 2.05	34.53 ± 2.05	0.002711	92
	6	125.14 ± 5.04	175.25 ± 4.95	0.0146	17
	7	24.27 ± 1.29	37.25 ± 2.07	0.002832	88
	8	57.85 ± 1.85	84.05 ± 1.32	0.006749	37
	9	52.37 ± 5.11	59.95 ± 3.25	0.00611	41
	10	2.5 0 ± 0.14	4.80 ± 0.36	0.000292	857
	11	22.57 ± 3.75	27.25 ± 4.01	0.002633	95
Cashew	1	25.38 ± 1.11	32.80 ± 0.97	0.00423	59
	2	3.52 ± 0.09	5.91 ± 0.15	0.000587	426
	3	4.80 ± 0.21	6.83 ± 0.28	0.0008	313
	4	5.81 ± 0.54	8.20 ± 0.78	0.000968	258
	5	4.85 ± 0.87	6.33 ± 0.45	0.000808	309
	6	8.74 ± 0.89	11.23 ± 0.92	0.001457	172
	7	7.72 ± 0.19	8.60 ± 0.48	0.001287	194
Almond	1	2.50 ± 0.21	6.21 ± 0.54	0.00025	1000
	2	7.81 ± 0.85	9.25 ± 0.77	0.000781	320
	3	9.43 ± 1.21	11.23 ± 0.92	0.000943	265
	4	2.30 ± 0.04	5.30 ± 0.08	0.00023	1087
Walnut	1	1.90 ± 0.32	4.90 ± 0.49	0.00019	1316
	2	3.70 ± 0.02	4.20 ± 0.03	0.000617	405
	3	2.80 ± 0.05	4.90 ± 0.07	0.000467	536

25 samples (13 %) contain aflatoxins equal to or above the quantification limit: 11 samples of pistachio, 7 samples of cashew, 4 samples of almond, and 3 samples of walnut.

**Fig. 1:**
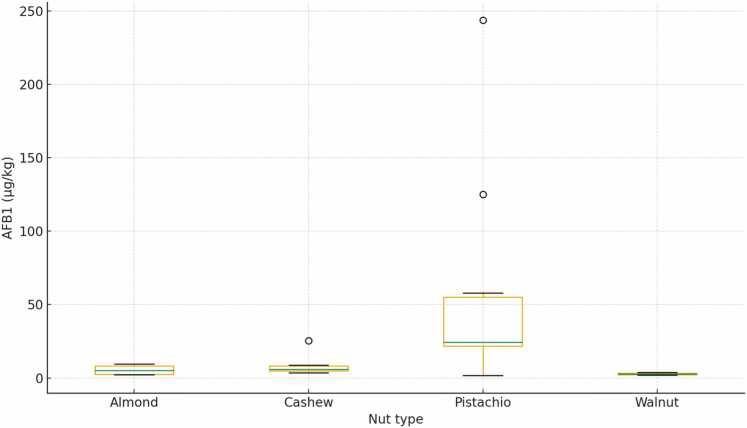
Aflatoxin B1 concentrations (µg/kg) in different types of nuts collected from local markets in Jordan. The boxes represent the interquartile range and the horizontal line inside each box indicates the median. High concentration of shown as individual points, illustrating variability and occasional high contamination levels among nut types.

Across positive samples, AFB1 (µg/kg) showed strong right-skewness. Medians and ranges were approximately for pistachio is 24.27 (1.79–243.83), cashew is 5.81 (3.52–25.38), almond is 5.06 (2.30–9.43), walnut is 2.80 (1.90–3.70) (Table 1, Fig. 1).

Nine samples of pistachio contain total aflatoxins and aflatoxin B1 above the maximum limits. Out of six samples of cashew containing aflatoxins, four samples have aflatoxin B1 above the limits, and two samples contain total aflatoxins above the maximum limit. Two samples of almonds have aflatoxin B1 above the maximum limits, while one sample has a total aflatoxin exceeding the limit. For walnut samples, all the samples have aflatoxins below the maximum limits.

The percentage of samples having aflatoxins is higher than that found by Salem and Ammar [19], in which the percentage of contaminated samples with aflatoxins was 3 %. The differences between the percentages may be due to different quantification methods used. In addition to that, the variations may be attributed to the hot, dry climate with very hot summers and mild winters that creates conditions favorable for the growth of fungi and secretion of their toxins [19], [5]. The average mean of aflatoxin B1 in pistachio, cashew, almond, and walnut samples was 54.7, 8.6, 5.5, and 2.8 respectively [19]. Based on the review performed by [10], the international average of aflatoxin B1 in pistachios, almonds, walnuts, among countries ranged from 0.00 in Spain to 233.60 µg/kg in Taiwan, 0.00 in Spain to 32.90 µg/kg in Cyprus, and 0.00 in Spain to 360 µg/kg in Morocco, respectively [10].

The results obtained revealed a wide variation in the levels of aflatoxins present in the nuts. This may be ascribed to various reasons including the type and amount of each nut, geographical influences during cultivation, harvesting time, processing and manufacturing conditions of the nuts, and the method of analysis, which can all influence the amount of aflatoxins within the nut samples [4].

### 3.3 Risk assessment of aflatoxin intake

The MOE approach was employed to evaluate the risk and public health implications associated with the consumption of nuts contaminated with aflatoxin B1. As shown in Table 1, all contaminated nut samples posed a potential risk, as the calculated MOE values of those samples were below 10,000, indicating a higher level of concern.

Based on the quantification of the aflatoxins within the samples, the EDI of it resulting from consuming these nuts could be determined. Based on daily consumption of the nuts as recommended, the EDI values of the aflatoxin B1 ranged from 0.2 to 28.4 ng/kg bw/day, assuming a body weight of 60 kg. The EDI of the current study is low compared to the EDI from other regions. The EDI was 0.93–2.45 ng/kg bw/day in Europe [12], from 3.5 to 180 ng/kg bw/day in Africa, from 0.3 to 53 ng/kg bw/day in Asia and at 2.7 ng/kg bw/day in the USA [6].

These EDI values resulted in MOE values that all samples were generally below 10000, ranging from 9.0 to 1316, indicating that the level of aflatoxins in nut samples possesses a high risk for human health. This range of MOE values was similar to MOE value that were calculated based on exposure dates in Europe, Africa, Asia, and the USA. The MOE values were 102–269 for Europe, 1–71 for Africa, 5–833 for Asia, and 780–1300 for USA. All MOE values worldwide, including this study’s results, showed a high risk of consumption of nuts contaminated with aflatoxins, even in low amounts near the detection limits [6].

In addition to that, other foodstuffs contaminated with aflatoxins increase the risk of public health. A study was conducted during 2021 in Jordan to assess the health risk associated with aflatoxin B₁ in poultry eggs and revealed that both adults and children are at a concerning health risk, with MOE < 10,000 [17].

The results reveal measurable levels of AB1 in nuts in Jordan market, with MOE values falling below the safety threshold of 10,000, indicating a significant public health concern [13]. Given the widespread consumption of nuts in Jordan, especially incorporated into traditional sweets, these results highlight the urgent need for regulatory interventions. Strengthening import surveillance and enforcing strict aflatoxin limits aligned with Codex Alimentarius standards [9] are critical steps toward minimizing exposure. Establishing a comprehensive monitoring program and implementing Hazard Analysis and Critical Control Points (HACCP) systems throughout the production and supply chain would further reduce fungal growth and toxin formation. Public awareness campaigns promoting safe storage, proper handling, and identification of mold-contaminated nuts are equally important. Ultimately, integrating these control strategies with ongoing surveillance and public education will help reduce aflatoxin-related health risks, strengthen food safety regulations, and protect consumer health in Jordan [10], [17].

## 4 Conclusion

The findings of this study demonstrate the presence of aflatoxins in nuts available in Jordanian markets, mainly in pistachios and cashews. MOE values for contaminated samples were consistently below the safety threshold, suggesting a public health concern. Given the widespread consumption of nuts in Jordan, often incorporated in traditional sweets, immediate action is needed to reduce aflatoxin exposure. This includes better storage and handling, stricter enforcement of regulatory limits, and public education campaigns. In addition to that, a surveillance and risk assessment plan should be conducted to develop comprehensive food safety strategies.

## CRediT authorship contribution statement

**Alajlouni Abdalmajeed:** Writing – review & editing, Writing – original draft, Validation, Supervision, Methodology, Conceptualization. **Al-Sakaji Amer:** Methodology, Formal analysis. **Abu-Hardan Mohammad:** Methodology, Formal analysis. **Dima Alkadri:** Writing – review & editing, Methodology, Conceptualization.

## Authors’ statement

Alajlouni AM conceived the idea, designed the study, collected samples, analyzed the results, collected literature, and wrote the manuscript. The methodology was performed by Dima Alkhadri, Abu-Hardan MS, and Al-Sakaji AA. All authors read and approved the final manuscript.

## Declaration of Competing Interest

The authors declare that they have no known competing financial interests or personal relationships that could have appeared to influence the work reported in this paper.

## Data Availability

Data will be made available on request.
