# Supplementary material for: Assessing aflatoxin exposure risk from imported nuts in the Jordan market
Source: Toxicol Rep. 2025 Dec 16;16:102190. doi: 10.1016/j.toxrep.2025.102190 (PMC12770948; doi:10.1016/j.toxrep.2025.102190)
Supplement: Supplementary file 1 — Supplementary material [file mmc1.docx]

## Table S1. Calibration data for aflatoxins

| Aflatoxin | Concentration Range (ng/mL) | Calibration Equation (y = mx + b) | Correlation Coefficient (r) |
| --- | --- | --- | --- |
| B₁ | 1–100 | y = 0.24305x + 0.11508 | 0.99970 |
| B₂ | 1–100 | y = 0.33696x – 0.01158 | 0.99963 |
| G₁ | 1–100 | y = 0.77639x – 0.25641 | 0.99975 |
| G₂ | 1–100 | y = 2.01273x – 0.61436 | 0.99968 |


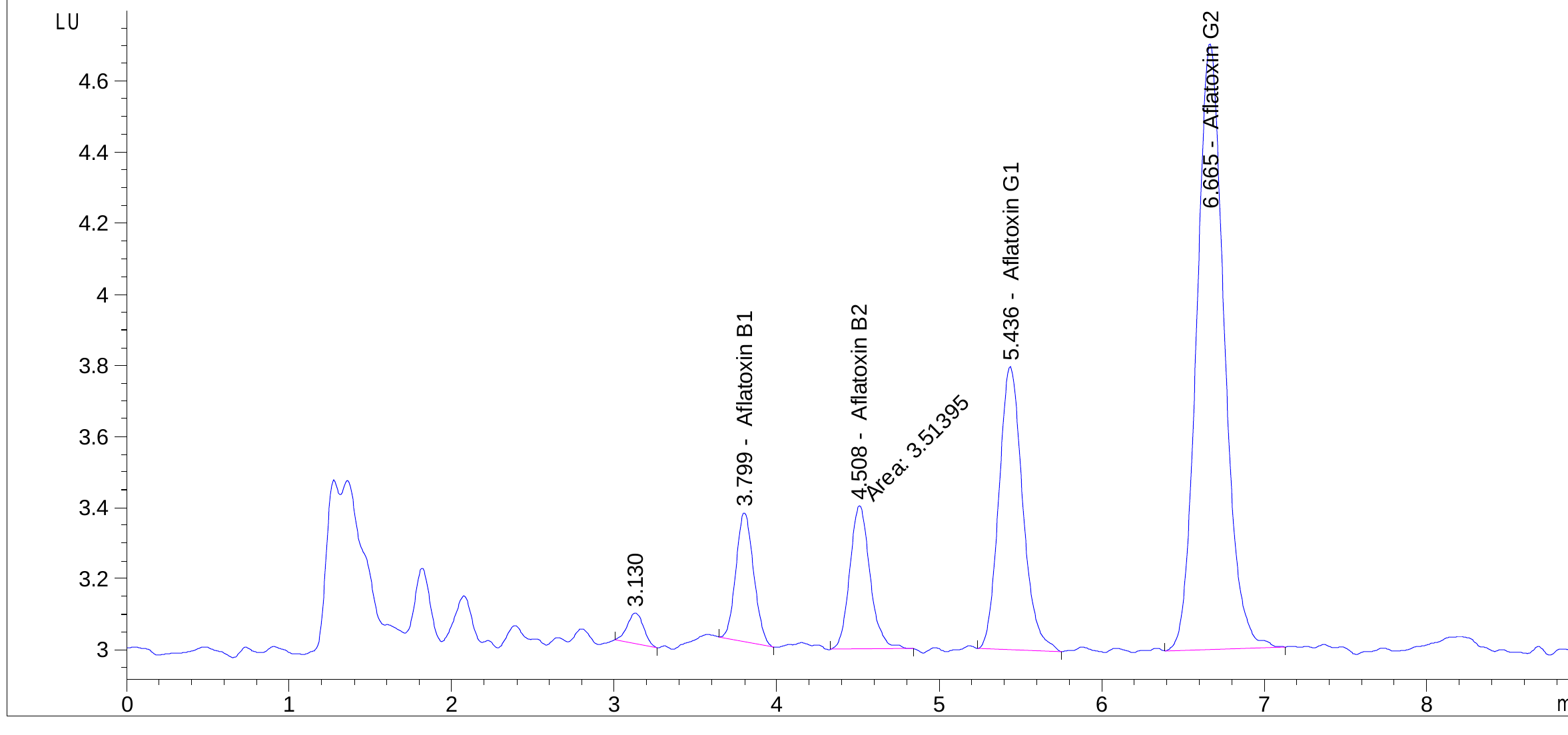


Figure S1. Matrix-matched chromatogram of aflatoxins.
